# Analysis of clinical characteristics and health resource costs in children hospitalised for injuries in southern Sichuan, China

**DOI:** 10.3389/fped.2023.1200886

**Published:** 2023-07-03

**Authors:** ZiWei Lu, YinSu Wang, Min Nie, JiaQin Li, YanHong Yu, Yuan Zhuang, Xiaoyan Mao, Xing Shen

**Affiliations:** ^1^ Department of Pediatrics, The Affiliated Hospital of Southwest Medical University, Luzhou, China; ^2^ Sichuan Clinical Research Center for Birth Defects, The Affiliated Hospital of Southwest Medical University, Luzhou, China

**Keywords:** clinical characteristics, children, hospitalization, injury, health resource costs

## Abstract

**Aim:**

To investigate the clinical characteristics and health resource costs among children hospitalised for injuries in southern Sichuan, China, and to provide guidance for prevention and treatment.

**Methods:**

We collected clinical data concerning children aged from 29 days to 18 years hospitalised for injuries from January 1, 2017, to December 31, 2021, retrospectively analysing the basic characteristics, evolution of injury characteristics over time, risk factors for events with adverse outcomes, and health resource costs.

**Results:**

Among 5,826 hospitalised children with injuries, males (63.6%), those in rural areas (40.3%), and adolescents (33.5%) were most commonly injured. Most injuries occurred at home (52.6%), and during summer. The most common injury types were falls, burns, road traffic injuries, poisoning, and foreign body injuries (32.0%, 17.9%, 13.6%, 8.8%, and 7.9%, respectively). After 2019, the proportion of intentional injuries among adolescent girls was significantly higher. Road traffic injuries most commonly led to poor clinical outcomes (95%CI: 5.39–31.51), followed by falls (95%CI: 2.20–10.67). Adolescents were at higher risk of poor prognosis. Injuries occurring in rural areas, adolescents, road traffic injuries, and falls cost high health resource.

**Conclusion:**

Injuries among children remain serious, with males and adolescents from villages predominantly affected. Attention should be paid to intentional injuries among adolescent females also. Targeted prevention and control measures for road traffic injuries and falls should be strengthened.

## Background

Due to socioeconomic development, mortality rates from infectious diseases have declined significantly (1). However, injuries are becoming an increasingly main threat to the health of children, because they are common causes of disability, and involve high healthcare costs (2). The Global Burden of Disease Study reported that over 3 million people died from unintentional injuries in 2015, of whom children aged <15 years comprised nearly 20% of the deaths (3). Furthermore, findings presented by the Chinese Children’s Safety Network showed that 200,000 children die from injuries each year (mortality rate, 67.13/100,000), which is 1.5 times that of South Korea and 2.5 times that of the United States, and it is increasing at an annual rate of 7%−10% (4). Tens of millions of children seek medical treatment for injuries, and some are left with lifelong disabilities that place a heavy burden on society, families, and individuals. Therefore, it is imperative to prevent and manage injuries to protect children’s health.

China is a vast country with complex geographical conditions, with each region having different manifestations of child injuries according to their living habits and environment (5, 6). Child injuries are also closely related to economic developmental levels associated changes, and resulting protective measures adopted (7–9).

The Southwest Medical University is located in the south of Sichuan, China, which bordering Yunnan, Guizhou, Sichuan, and Chongqing provinces, has diverse topography, numerous ethnic groups, different living habits, and varying economic developmental levels. While the characteristics of child injuries in this region compared with other regions of China might differ, and evolve with socioeconomic development, no relevant study to date has reported on child injuries within this region. Meanwhile, our hospital is the region’s most important medical centre; thus, its inpatient data is partially representative of the population to some extent. This study aimed to retrospectively investigate the clinical characteristics and changes over time in child injuries, evaluate the possible risk factors for different injury outcomes, and determine relevant injury-related health resource costs, to provide evidence-based guidance on future prevention and treatment of child injuries within southern Sichuan region, where our hospital is located.

## Methods

We retrospectively investigated clinical data concerning children aged between 29 days and 18 years and hospitalised for injuries at the affiliated hospital of Southwest Medical University from January 1, 2017 to December 31, 2021. If a paediatric patient was admitted multiple times for sequelae owing to an injury, only the first injury-related case was recorded. If a paediatric patient had a combination of multiple types of injuries, the most predominant type of injury was recorded. Paediatric patients with a hospital stay <24 h, missing hospitalisation data, and an unknown prognosis were excluded. Diagnosis codes were entered according to the WHO International Classification of Diseases (ICD-10) and included the following 14 types of injury: road traffic injuries, falls, burns, sharps injuries, bruises, smash injuries, crush injuries, blast injuries, bites, electrocution, drowning, poisoning, foreign body injuries, and others/unknown. Age was divided into five groups according to *Paediatrics* (9th Edition) as follows: infancy (28 days-<1 year), early childhood (1-<3 years), preschool (3-<6 years), school age (6-<10 year), and adolescence (10-<18 years). Places of residence are divided by the state administrative departments into villages, towns, counties, and urban areas.

We analysed data concerning the basic characteristics of the paediatric patients (sex, age, place of residence, location of injury, and season), evolution of injury characteristics over time, risk factors for events with adverse outcomes (defined as one or more organ dysfunction or death occurs), and medical resource costs.

SPSS version 26.0 (IBM Corp., Armonk, NY, USA) was used for statistical analyses. Count data are expressed as a proportion (%). Furthermore, measurement data conforming to a normal distribution are expressed as mean ± standard deviation (*X̄ ± S*), whereas measurement data with skewed distribution are expressed as median (M, interquartile range, P_25_–P_75_). A chi-square test was used to explore the significance of differences between groups, and Fisher’s exact test was used when the conditions for a chi-square test were not met. Additionally, a *t*-test was used for normally distributed data, and a Kruskal-Wallis rank sum test was used for not normally distributed data. A Spearman’s rank correlation coefficient was used to analyse the correlation between different indicators, and a multinomial logistic regression model was used to evaluate potential risk factors. Statistical significance was set at a *P-*value < 0.05.

## Results

### Basic characteristics of paediatric patients hospitalised for injuries

In total, 5,826 paediatric patients with injuries were admitted to our hospital from 2017 to 2021, most of which them were male (63.6%), adolescent (33.5%), and belonging to rural areas(village-based;40.3%). The most common location of injuries was at home (52.6%), and the five most common types of injuries were falls (32%), burns (17.9%), road traffic injuries (13.6%), poisoning (8.8%), and foreign body injuries (7.9%) (Table 1). Injuries were more common during summer, reaching a peak in July (Supplementary Figure S1).

**Table 1:** Basic characteristics of children hospitalized for injuries from 2017 to 2021.

	Number of cases	Constituent ratio (%)
Total	5,826	
**Gender**		
Male	3,708	63.6
Female	2,118	36.4
**Age group**		
Adolescent	1,949	33.5
Early childhood	1,422	24.4
Preschool age	1,208	20.7
School age	1,010	17.3
Infancy	237	4.1
**Places of residence**		
Village	2,346	40.3
Town	1,301	22.3
County	1,185	20.3
Urban	994	17.1
**Location of injury**		
Home	3,066	52.6
Public place	2,399	41.2
School	361	6.2
**Types of injury**		
Falls	1,865	32.0
Burns	1,040	17.9
Road traffic injuries	790	13.6
Poisoning	512	8.8
Foreign body injuries	463	7.9
Bites	268	4.6
Sharps injuries	204	3.5
Bruises	174	3.0
Others or unknown	137	2.3
Smash injuries	124	2.1
Crush injuries	109	1.9
Blast injuries	56	1.0
Drowning	55	0.9
Electrocution	29	0.5

### Changes the characteristics of patient for injuries along the study timeline

#### Proportion of injured patients

The number of resident populations in this region from 2017 to 2021 is 4,214,000, 4,226,000, 4,244,000, 4,256,000 and 4,259,000. The proportion of paediatric patients hospitalised for injuries was relatively stable in 2017 (34.36 per 100,000) and 2018 (31.52 per 100,000), but decreased from 2019 to 2021 (average proportion of injured patients for these three years, 23.88 per 100,000; Supplementary Figure S2). In addition, we found that the number of paediatric patients hospitalised for injuries was the highest in village areas (*χ*^2 ^= 34.38, *P *< 0.001), followed by town, county and urban (Supplementary Figure S3). While the gender gap is narrowing, but more boys were hospitalised (Supplementary Figure S4).

#### Trend of common types of injuries

**Figure 1:**
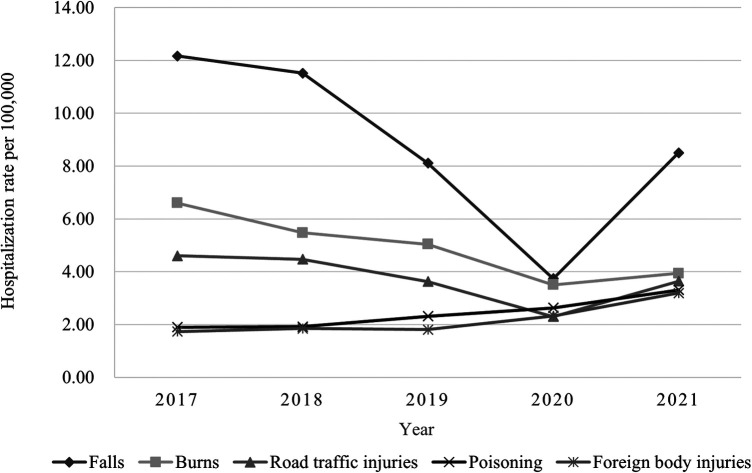
Incidence rate of common injury types from 2017 to 2021.

Although falls represented the most common type of injury across all study years, there was an overall decreasing trend (*χ*^2 ^= 66.71, *P* < 0.001). Meanwhile, burns showed a significant decrease too (*χ*^2^ = 15.45, *P *< 0.001). Traffic injuries ranked third among different types of injuries in four of the five years examined. Poisoning (*χ*^2^ = 78.56, *P *< 0.001) and foreign body injuries (*χ*^2 ^= 72.64, *P* < 0.001) were a yearly increasing trend (Figure 1).

#### Mode of injury

**Figure 2:**
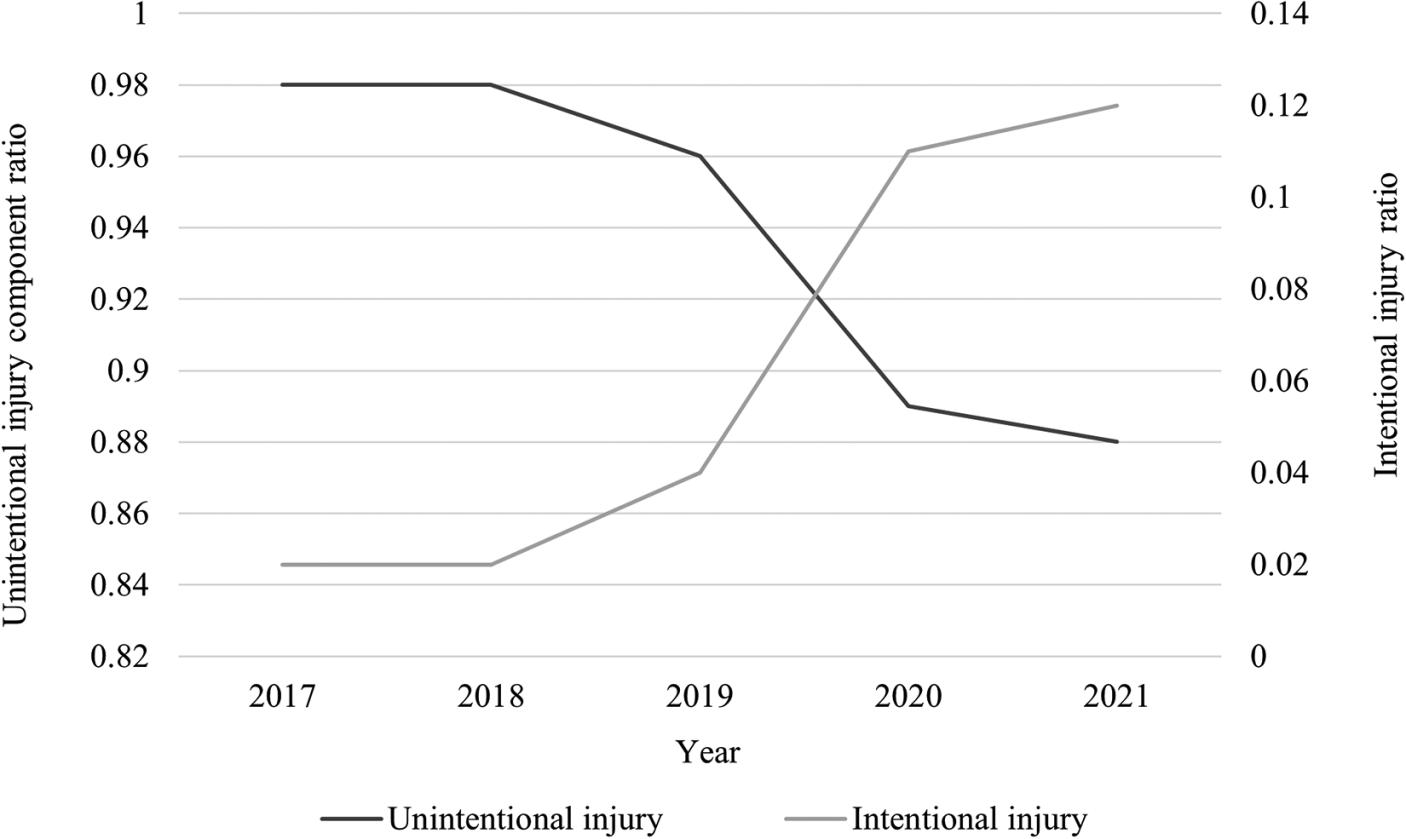
Annual changes in injury modes of children hospitalized due to injury.

The proportion of intentional injuries started to rise annually from 2018, especially after 2019 (Figure 2). Self-inflicted injuries, accounting for 88.3% of intentional injuries, were more common in female adolescent patients from villages, often occurred at home, and were mostly due to ingestion of drugs or poisonous substances (Supplementary Table S1).

### Analysis of risk factors for events with adverse outcomes

Among different types of injuries, road traffic injuries (odds ratio [OR] 13.03; 95% confidence interval [CI], 5.39–31.51) and falls (OR, 4.85; 95% CI, 2.20–10.67) were the main risk factors for adverse outcomes in paediatric patients hospitalised for injuries. Infants and adolescents were at a higher risk for adverse outcomes than other age groups (Table 2).

**Table 2:** The most common injury type outcome risk factors.

	*P*	OR	95% CI
**Types of injury**	<0.001		
Road traffic injuries	<0.001	13.03	(5.39, 31.51)
Falls	<0.001	4.85	(2.20, 10.67)
Poisoning	0.07	1.87	(0.96, 3.66)
Foreign body injuries Burns	0.17	2.06 1.00	(0.74, 5.75)
**Age group**	<0.001		
Infancy	0.10	1.72	(0.91, 3.27)
Early childhood	0.02	0.53	(0.31, 0.91)
Pre-school age	0.002	0.46	(0.29, 0.74)
School age Adolescent	0.008	0.52 1.00	(0.32, 0.85)
**Location of injury**	0.20		
Home	0.41	1.25	(0.74, 2.11)
School Public place	0.16	0.47 1.00	(0.16, 1.35)
**Places of residence**	<0.001		
Village	0.09	1.62	(0.93, 2.80)
Town	0.24	1.46	(0.77, 2.77)
County Urban	<0.001	3.21 1.00	(1.88, 5.51)

### Medical resource costs

#### Average hospitalisation costs

The average annual hospitalisation costs for paediatric patients hospitalised for injuries were high across the study period, fluctuating between $1,847.07 and $2,703.97, with an overall increasing trend after 2018 (Supplementary Figure S5).

#### Median hospitalisation costs for different types, age group and places of residence

The overall distribution of median hospitalisation costs was not identical across different types of injuries, the age groups and the places of residence due to injury (*P *< 0.001*)*. In sorts of types, the highest cost was road traffic injuries, followed by falls and drowning (Table 3). Age was positively correlated with the median hospitalisation costs (*r*_s _= 0.15, *P *< 0.001), and the highest costs was associated with adolescence (Supplementary Table S2). In village areas, the median hospitalisation cost was the highest and far exceeded that in urban areas (Supplementary Table S3).

**Table 3:** Comparison of median hospitalization costs of different injury types in children hospitalized due to injury (dollar).

Types of injury	Hospitalisation costs [M (P_25_, P_75_)]
Road traffic injuries	1783.20 (4713.61, 4458.85)
Falls	1670.39 (743.01, 2967.86)
Drowning	1421.93 (804.14, 2119.43)
Crush injuries	1385.70 (1001.51, 1727.68)
Sharps injuries	1353.27 (946.31, 1811.92)
Smash injuries	1330.63 (648.79, 2964.87)
Blast injuries	1225.78 (790.43, 2157.71)
Others or unknown	1151.88 (659.54, 1683.05)
Burns	1110.00 (689.45, 2081.55)
Electrocution	896.36 (491.94, 1870.73)
Poisoning	853.73 (531.09, 1779.00)
Bruises	745.64 (399.23, 1296.23)
Foreign body injuries	647.70 (528.67, 1196.07)
Bites	528.88 (397.50, 769.59)

*H = *565.03, *P *< 0.001.

## Discussion

In this study, we found that childhood injuries occurred predominantly in boys, in rural areas, at home, during spring and summer with a peak in July. These findings are consistent with those reported in previous studies in other regions of China (10, 11). One explanation may be that men tend to be more physically active and thus may be more prone to injuries. The other explanation could be that, our hospital is located in the southernmost region of China, has a notably warmer weather in spring and summer, with the summer vacation being in July. These environmental factors might contribute to a higher occurrence of unintentional injuries in July. However, the most common types of injuries in our region were falls, burns, road traffic injuries, poisoning, and foreign object injuries. These findings are not entirely consistent with that of domestic and foreign regions (12–14), which showed that burns and poisonings being less frequent. This variation may be due to our region have many mountainous areas, cold winters, and residents relying on burning charcoal for heat. In addition, the village areas are extensive and mainly agriculture-oriented; thus, the use of pesticides and herbicides is higher than that in industrial areas, which may explain the higher number of children being poisoned. Our findings also showed that more adolescent patients were hospitalised for injuries. Our review of the previous literature found that the age group with the highest prevalence of injuries predominately involved older children (15, 16). Contrary to popular belief, adolescents tend to be more vulnerable to injuries than younger children. This is because guardians may assume that adolescents have a better sense of safety than younger children, and neglect supervising them; However, their awareness of safety is actually immature. Therefore, attention should be paid to this population in relation to injury prevention.

In terms of the changes along the timeline, we found that our hospital had an average of 27.50 per 100,000 paediatric patients hospitalised for injuries every year, male children consistently predominated, and the proportion of injuries occurring in village areas consistently remained high. This suggests that the risk of child injuries is significant and that their prevention and management should be strengthened, particularly in male children in village areas. Interestingly, the number of injured patients was a slight decreasing in 2019–2020, which we presumed to be a direct consequence of the strict preventative measures taken during the COVID-19 pandemic. During that period, children stayed at home with their guardians for a longer time, had a significant reduction in social activities, and may have been better cared for; thus, fewer injuries occurred. This also proves that the injuries are preventable as long as the three factors of injury prevention are well taken into account, the factors affecting injury proposed by John Gordon (17): host, pathogenic, and environmental.

In this study, the proportion of intentional injuries was significantly higher after 2019. Further analysis indicated that those with intentional injuries predominantly comprised adolescent girls in village areas, most took poisons or medications to intentionally harm themselves. This result is consistent with findings reported by Zhu et al. (18). Intentionally injured older children may often have depression or be in intense emotional conflict with their guardians, peers, or teachers (18). Data show that the rate of poisoning in children due to exposure to antidepressants has increased to 1,793 cases per year (5.8%/year) over the past ten years, leading to events with extremely serious adverse prognoses (19). Depression and anxiety, which are mood disorders, were once considered ‘urban diseases’, more likely to occur in economically developed areas. However, owing to recent rapid economic development in China, regions which had previously been economically underdeveloped are now presenting with increasing numbers of individuals with depression and anxiety, suggesting that extra attention should be paid to in future.

This study also showed that road traffic injuries was consistently the third most common type and a most likely risk factor to cause adverse outcomes, consistent with studies in other regions in China (20), Australia (21) and Canada (22). This could be linked to increasing vehicle ownership rates have resulted in a higher incidence rate of road traffic injuries (23), which is more likely to lead to multiple organ function impairment. China’s legislation on the protection of minors stipulates that vehicles should be equipped with child safety seats (CSSs) (24). However, most parents consider CSSs optional and prefer to hold their children on their laps, considering this to be safer (25), particularly in western areas of China. So, stricter implementation of this law concerning the use of CSSs is needed. Furthermore, the falls were consistently the most common cause of injury and the second most likely factor leading to a poor prognosis. Adolescents from villages had a higher number of poor prognostic outcomes, consistent with previous research results (26, 27). Indicating that road traffic injuries, falls, and adolescents from villages should be targeted in terms of injury prevention in the future.

The average annual hospitalisation costs of child injuries in our hospital were $2312.22. Of all injuries, road traffic injuries and falls were the most costly injuries, in line with Australia (21) and India (28). Compared with other diseases, the costs of child injuries are higher than that of asthma ($1723), acute upper respiratory infections ($2057) and influenza ($2072), comparable to bronchiolitis ($2602) and pneumonia ($2929) (29). And it’s significantly higher than that in Northwest China 10 years ago ($166) (30), indicating that the costs of child injuries are constantly increasing. In order to reduce the financial burden, more measures should be taken to prevent children from injury in the future.

## Conclusion

Child injuries continue to be a serious issue, with males and adolescents from villages predominantly affected. Further attention should also be paid to intentional injuries among female adolescents. Road traffic injuries and falls were the most harmful types of injuries in terms of occurrence proportion, clinical outcomes, and medical resource costs; thus, prevention and management strategies should be strengthened.

## Data Availability

They contain information that could compromise the privacy of the child, and the data cannot be made public. Requests to access these datasets should be directed to ZL, 603243561@qq.com.
