# Supplementary material for: Analysis of clinical characteristics and health resource costs in children hospitalised for injuries in southern Sichuan, China
Source: Front Pediatr. 2023 Jul 3;11:1200886. doi: 10.3389/fped.2023.1200886 (PMC10351037; doi:10.3389/fped.2023.1200886)
Supplement: Supplementary file 1 [file Table1.docx]

sTable 1. Distribution characteristics of injury types in hospitalized children due to intentional injury [n (%)]

|  | Self-inflicted injuries  (n=286) | Injury caused by others (n=38) |
| --- | --- | --- |
| **Gender** |  |  |
| Male | 70(24.5%) | 35(92.1%) |
| Female | 216(75.5%) | 3(7.9%) |
| **Age stage**  Early childhood | 0(0.00%) | 1(2.6%) |
| Adolescent | 286(100%) | 37(97.4%) |
| **Places of residence** |  |  |
| Village  Town | 156(54.6%)  39(13.6%) | 11(28.9%)  8(21.1%) |
| County | 40(14.0%) | 12(31.6%) |
| Urban | 51(17.8%) | 7(18.4%) |
| **Location of injury** |  |  |
| Home | 254(88.8%) | 2(5.3%) |
| School | 29(10.1%) | 5(13.1%) |
| Public place | 3(1.1%) | 31(81.6%) |
| **Types of injury** |  |  |
| Poisoning | 250(87.4%) | 0(0.0%) |
| Falls | 14(4.9%) | 0(0.0%) |
| Others or unknown | 22(7.7%) | 38(100%) |
