# Supplementary material for: Analysis of clinical characteristics and health resource costs in children hospitalised for injuries in southern Sichuan, China
Source: Front Pediatr. 2023 Jul 3;11:1200886. doi: 10.3389/fped.2023.1200886 (PMC10351037; doi:10.3389/fped.2023.1200886)
Supplement: Supplementary file 2 [file Table2.docx]

sTable 2. Comparison of median hospitalization costs among age groups of children hospitalized due to injury (dollar)

| Age group | hospitalization costs [M (P_25_, P_75_)] |
| --- | --- |
| Infancy | 807.78(397.38, 1704.86) |
| Early childhood | 1024.85(611.97, 1860.68) |
| Preschool age | 1139.88(598.80, 2243.33) |
| School age | 1201.08(606.92, 2422.06) |
| Adolescent | 1456.65(705.54, 3459.48) |

Note: *H=*135.08, *P*<0.001.
