# Supplementary material for: Analysis of clinical characteristics and health resource costs in children hospitalised for injuries in southern Sichuan, China
Source: Front Pediatr. 2023 Jul 3;11:1200886. doi: 10.3389/fped.2023.1200886 (PMC10351037; doi:10.3389/fped.2023.1200886)
Supplement: Supplementary file 3 [file Table3.docx]

sTable 3. Comparison of median hospitalization costs of children hospitalized due to injuries in different places of residence (dollar)

| Places of residence | Hospitalisation costs [M (P_25_, P_75_)] |
| --- | --- |
| Village | 9122.09(4719.81, 17715.12) |
| Town | 8026.08(4384.82, 15519.38) |
| County | 8235.55(4100.48, 16779.87) |
| Urban | 6025.48(3420.81, 11707.55) |

Note: *H*=119.45, *P*<0.001
