# Supplementary material for: Analysis of clinical characteristics and health resource costs in children hospitalised for injuries in southern Sichuan, China
Source: Front Pediatr. 2023 Jul 3;11:1200886. doi: 10.3389/fped.2023.1200886 (PMC10351037; doi:10.3389/fped.2023.1200886)
Supplement: Supplementary file 4 [file Table4.docx]

sTable 4. The outcome distribution of injury [n (%)]

| Argument | Full recovery (n=5559) | Adverse outcome (n=267) | *χ^2^* | *P* |
| --- | --- | --- | --- | --- |
| **Types of injury** |  |  | 94.93 | <0.001 |
| Road traffic injuries | 722(91.4%) | 68(8.6%) |  |  |
| Falls | 1798(96.4%) | 67(3.6%) |  |  |
| Poisoning | 502(98.0%) | 10(2.0%) |  |  |
| Foreign body injuries  Burns | 456(98.5%)  1032(99.2%) | 7(1.5%)  8(0.8%) |  |  |
| **Age group** |  |  | 30.14 | <0.001 |
| Infancy | 201(93.1%) | 15(6.9%) |  |  |
| Early childhood | 1191(98.2%) | 22(1.8%) |  |  |
| Pre-school age | 1015(97.4%) | 27(2.6%) |  |  |
| School age Adolescent | 762(96.8%) | 25(3.2%) |  |  |
| Adolescent | 1342(95.0%) | 70(5.0%) |  |  |
| **Location of injury** |  |  | 42.49 | <0.001 |
| Home | 2560(97.9%) | 54(2.1%) |  |  |
| School | 240(98.4%) | 4(1.6%) |  |  |
| Public place | 1711(94.4%) | 101(5.6%) |  |  |
| **Places of residence** |  |  | 31.51 | <0.001 |
| Village | 1766(97.0%) | 55(3.0%) |  |  |
| Town | 774(97.2%) | 22(2.8%) |  |  |
| County | 999(94.0%) | 64(6.4%) |  |  |
| Urban | 972(98.2%) | 18(1.8%) |  |  |
