# Supplementary figures and images for: Analysis of clinical characteristics and health resource costs in children hospitalised for injuries in southern Sichuan, China
Source: Front Pediatr. 2023 Jul 3;11:1200886. doi: 10.3389/fped.2023.1200886 (PMC10351037; doi:10.3389/fped.2023.1200886)

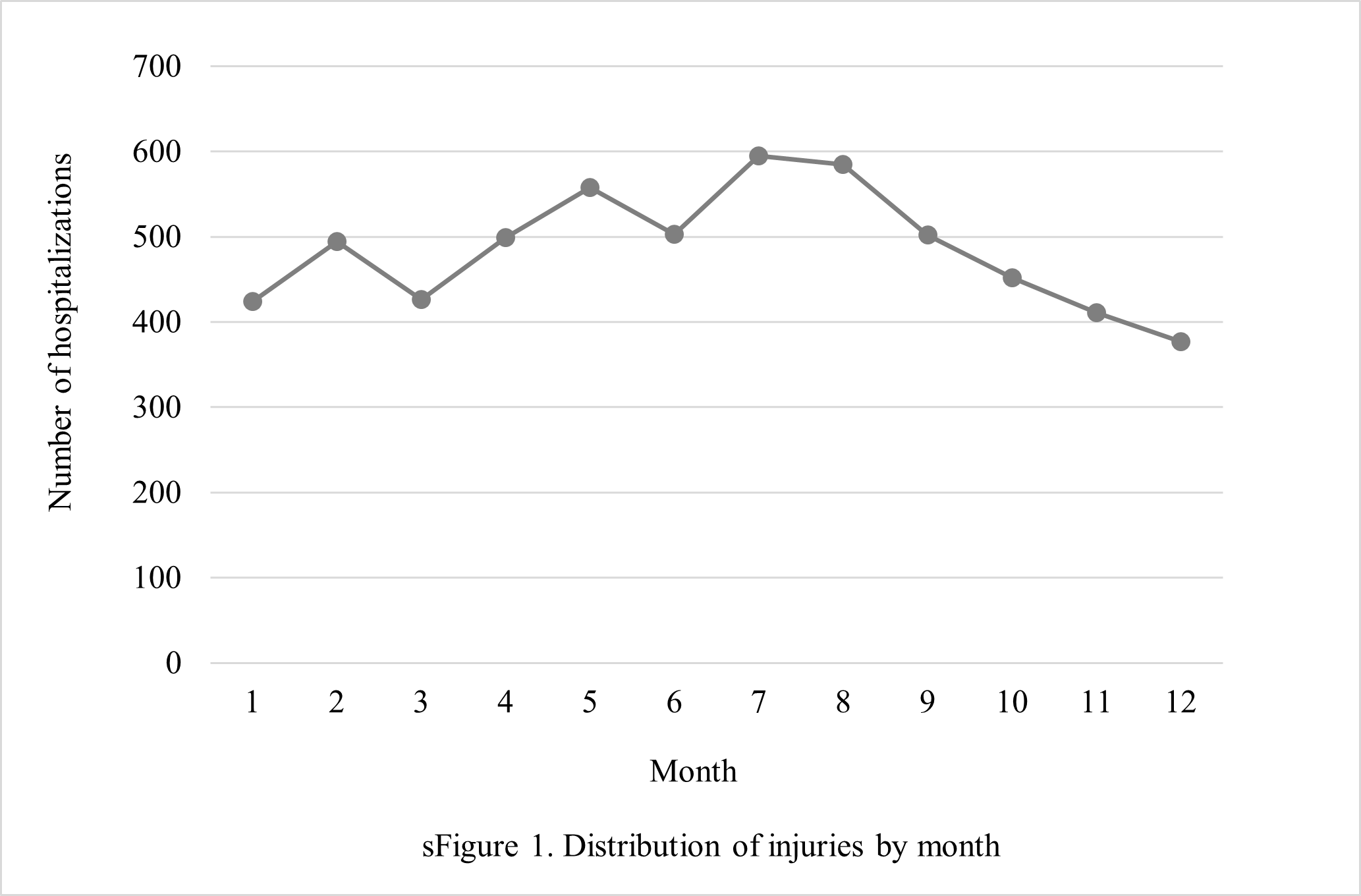

Supplement: Supplementary file 5 [file Image1.tif]

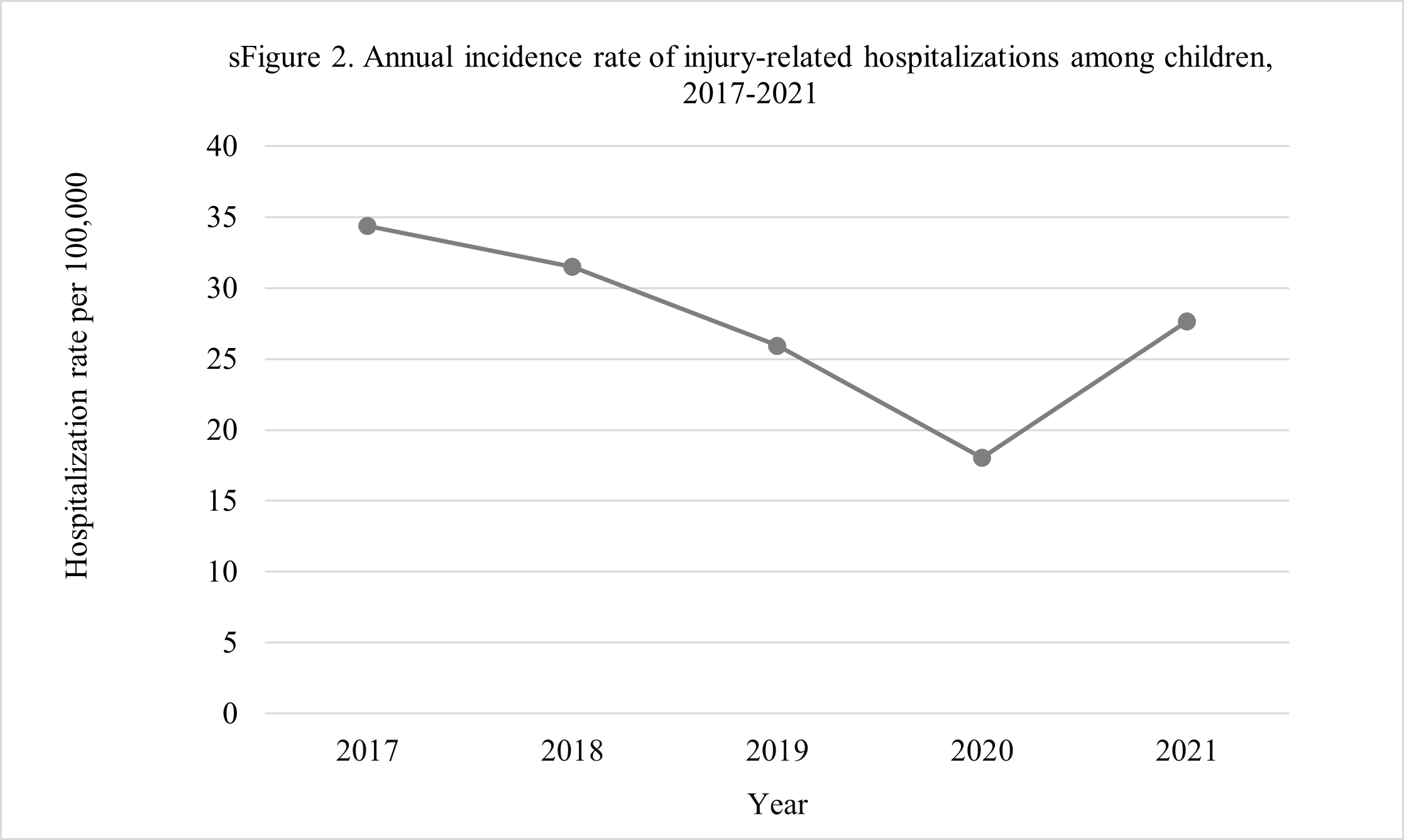

Supplement: Supplementary file 6 [file Image2.tif]

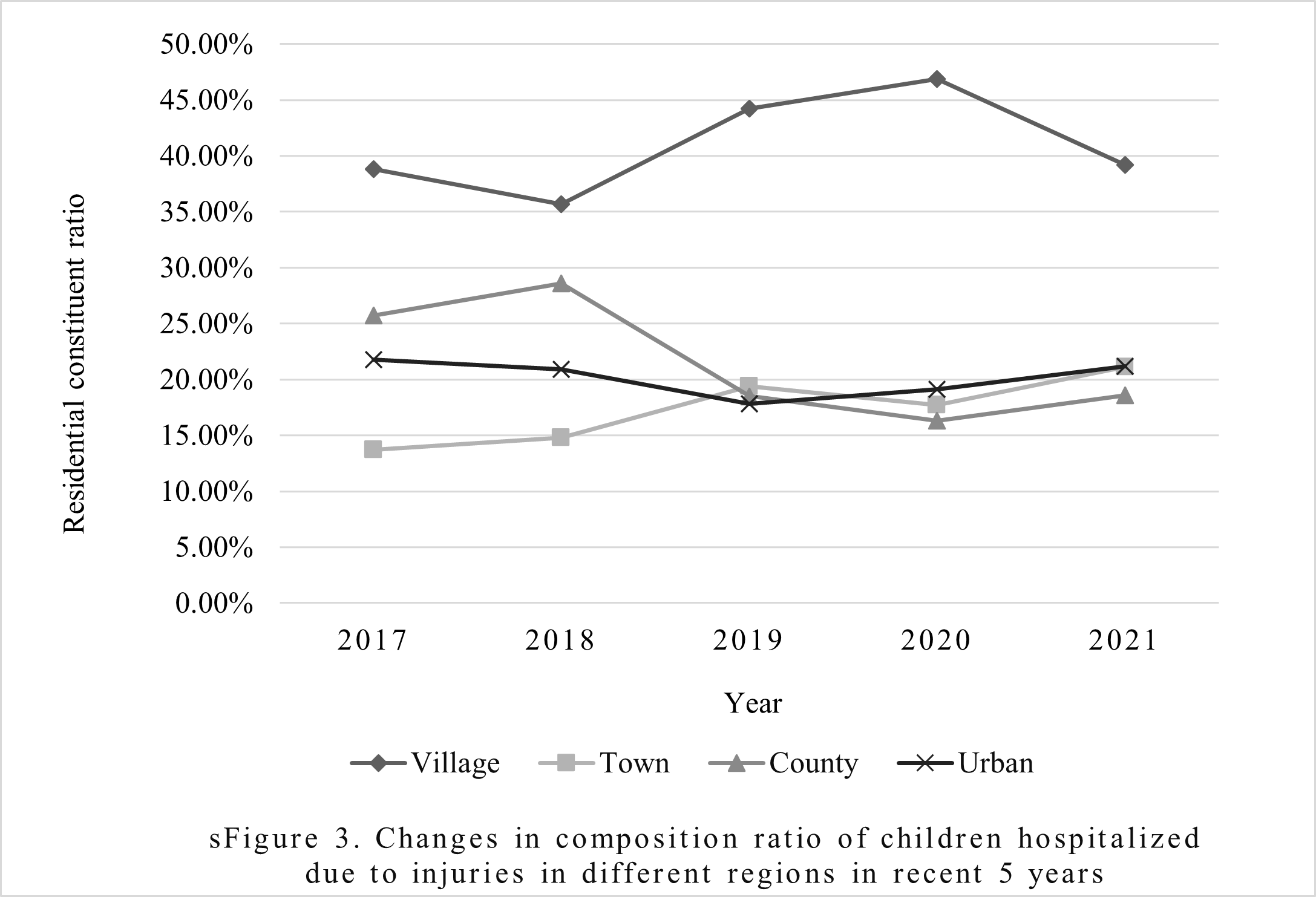

Supplement: Supplementary file 7 [file Image3.tif]

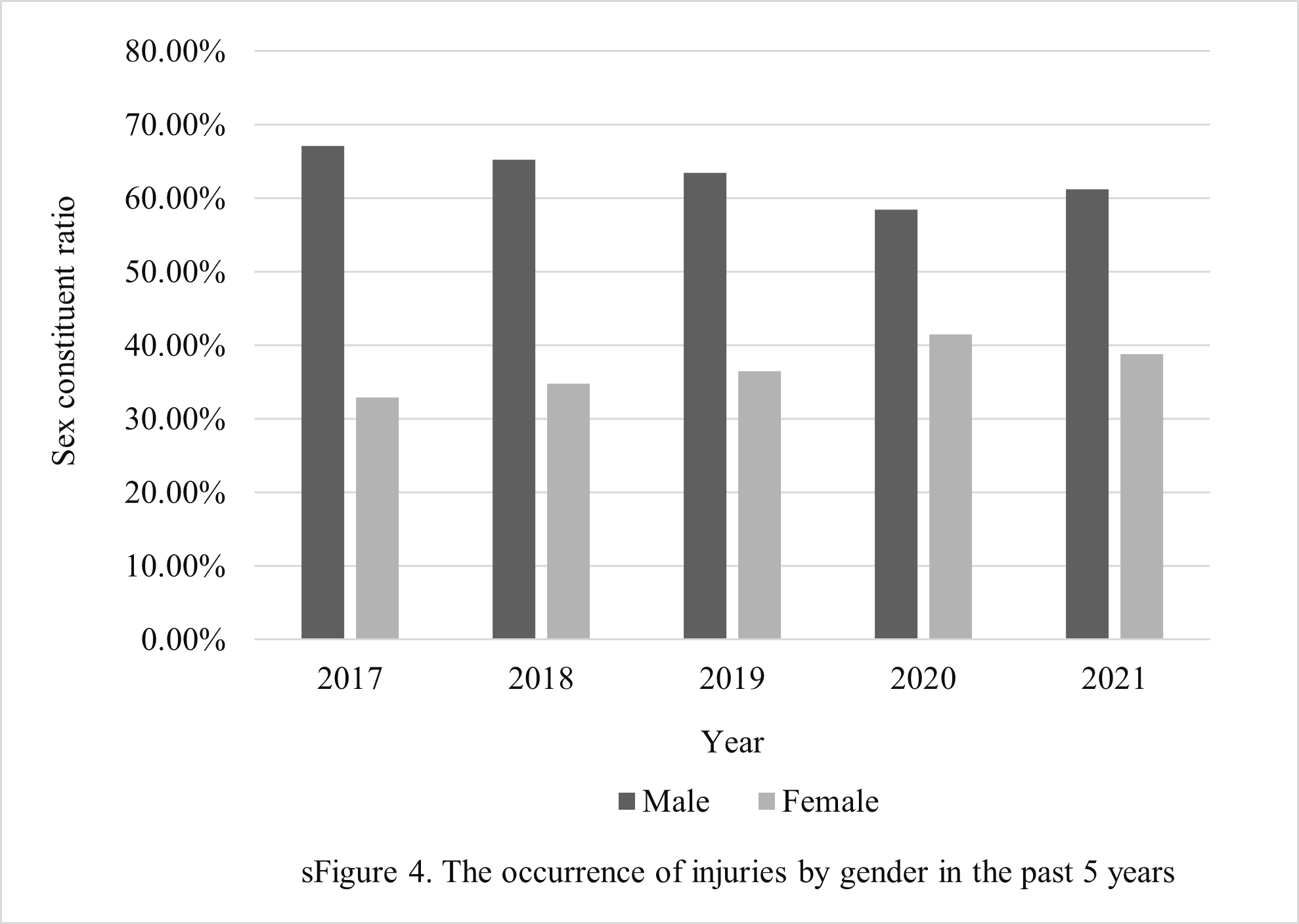

Supplement: Supplementary file 8 [file Image4.tif]

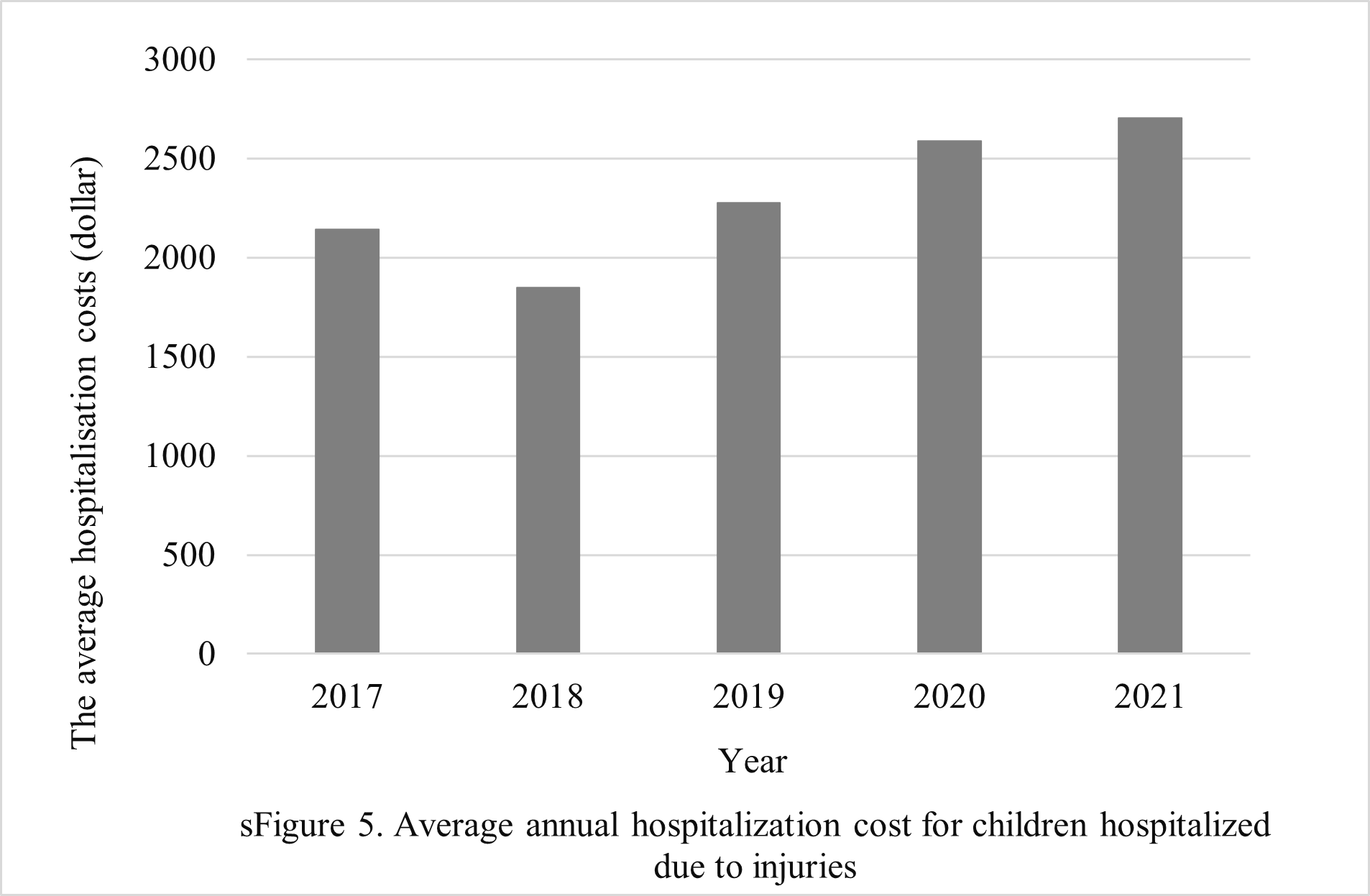

Supplement: Supplementary file 9 [file Image5.tif]
